# Effects of cognitive workload during motor-cognitive training on executive function, dual-task performance, and posture control: a RCT among middle-aged and older adults

**DOI:** 10.3389/fnagi.2026.1861129

**Published:** 2026-08-05

**Authors:** Yi Zhao, Xiaojing Li, Zhengyue Loumeng, Qi Li, Wei Sun, Xuewen Tian, Binghong Gao, Ran Li

**Affiliations:** 1 School of Postgraduate Education, Shandong Sport University, Jinan, Shandong, China; 2 Department of Science and Technology, Shandong Sport University, Jinan, Shandong, China; 3 School of Athletic Performance, Shanghai University of Sport, Shanghai, China; 4 School of Health Science, Springfield College, Springfield, MA, United States; 5 School of Exercise and Health, Shandong Sport University, Jinan, Shandong, China; 6 School of Health Sciences and Sports, Macao Polytechnic University, Macao, China

**Keywords:** cognitive workload, dual-task performance, executive function, interactive motor-cognitive training, older adults, postural control

## Abstract

**Background:**

Falls are a major concern in geriatric health, and impairments in executive function (EF), gait, and postural control are important fall-related risk factors. Interactive motor-cognitive training (IMCT) has shown promise as an intervention to mitigate these risks. This study aims to assess the acute effects of IMCT programs with three levels of cognitive load (high, medium, and low) on EF, spatio-temporal gait parameters, and postural control in middle-aged and older adults.

**Methods:**

In this single-blind, parallel-group randomized controlled trial, 42 participants (58.1 ± 9.9 years) were randomly allocated to a low cognitive load group (LCG), medium cognitive load group (MCG), or high cognitive load group (HCG). Each group completed a 30-min IMCT session using the SpeedCourt system. EF, postural control, and single/dual-task gait performance were assessed before and after the intervention.

**Results:**

Multivariate analysis showed significant between-group differences in EF (Pillai V = 0.804, *p* = 0.015, *η_p_^2^* = 0.402) and gait performance (Pillai V = 1.201, *p* < 0.001, *η_p_^2^* = 0.601); no significant changes were observed in postural control (Pillai V = 0.543, *p* = 0.323, *η_p_^2^* = 0.271). The MCG showed greater improvement in Stroop neutral reaction time than the LCG and HCG, while 2-back accuracy improved relatively more in the LCG. The MCG also showed greater improvement in dual-task gait velocity, whereas the LCG showed a greater increase in correct responses during dual-task walking. Discriminant function analysis identified Stroop neutral reaction time, 2-back accuracy, gait velocity, and correct responses as the most sensitive variables.

**Conclusion:**

A single session of IMCT with different cognitive loads was associated with immediate changes in selected EF and dual-task gait outcomes in middle-aged and older adults. Moderate cognitive load appeared most favorable for processing-speed-related Stroop performance and dual-task gait velocity, whereas low cognitive load was associated with greater gains in dual-task correct responses.

## 1 Introduction

The World Health Organization (WHO) has reported that the incidence rate of falls among the elderly aged 65 and above ranges from 28 to 35%, escalating to 32 to 42% for those over 70 years old (Yoshida, 2007). Falls are a significant concern in geriatric health, often leading to a series of adverse outcomes. These outcomes include physical injury, loss of independence, and negative psychosocial reactions (Schoene et al., 2014). The physical and psychological consequences of falls not only cause considerable distress to the elderly but also impose a substantial burden on their families in terms of daily care (Rubenstein, 2006).

Falls have a multifactorial etiology, shaped by the interaction of neuromuscular impairment, gait and balance dysfunction, sensory deficits, comorbidity, medication effects, and environmental hazards (Montero-Odasso et al., 2022). Among the intrinsic risk factors, the cognitive decline associated with aging has emerged as a critical factor in predicting falls among the elderly. Notably, executive function (EF), a higher-order cognitive function, is particularly vulnerable to age-related impairment. Evidence indicates that subtle declines in executive function, processing speed, and divided attention begin to manifest much earlier, typically during middle age, around 45 years old (Singh-Manoux et al., 2012). EF can influence falls through multiple pathways. For instance, a reduction in attention can lead to a decrease in balance ability (Dault et al., 2001) and the manifestation of gait abnormalities (Holtzer et al., 2006). Moreover, central processing and integration dysfunction, as well as postural response executive dysfunction (Caetano et al., 2019), also play a role. The attenuation of cognitive function in the elderly, compounded by the increased cognitive demands for postural control, poses greater challenges in maintaining postural stability. As a result, when the elderly engage in dual tasks, such as walking and talking simultaneously, their motor stability deteriorates, thereby heightening the risk of falls (Jehu et al., 2017). Importantly, this cognitive contribution to dual-task instability is not confined to old age. In a population-based cohort, dual-task gait cost began to rise from around 54 years of age and beyond this point interindividual differences in cognitive function accounted for a substantial share of the age-related decline (Zhou et al., 2023). Consistent with this, generalized slowing and reduced response preparation are already detectable by midlife and become more pronounced when an additional task raises attentional demand (Wolkorte et al., 2014). To address this issue and prevent falls across middle and older age, intervention strategies typically focus on resolving the problems encountered during dual-task execution, especially those common daily tasks like walking and talking or making visuospatial judgments, which are of utmost importance in fall prevention.

In response to this, the motor-cognitive dual-task training paradigm has emerged. By simultaneously challenging mobility while engaging attention and executive control, this approach targets the functional demands of everyday situations (Hillel et al., 2019; Yogev-Seligmann et al., 2008). Notably, because gait and postural control may require greater attentional resources when a secondary task is introduced, dual-task paradigms are particularly useful for revealing age-related cognitive-motor interference and task-related reductions in stability (Woollacott and Shumway-Cook, 2002). Because such interference emerges gradually across the adult lifespan rather than abruptly in old age, these paradigms are increasingly relevant to middle-aged as well as older adults, particularly as a means of acting before fall-related decline becomes established. Interactive motor-cognitive training (IMCT), exemplified by exergames and virtual reality games, constitutes a distinctive form of motor-cognitive training. It necessitates the simultaneous execution of two tasks and allows participants to engage with the computer interface through large muscle movements, while receiving immediate visual, auditory, or tactile feedback from the training system (Pichierri et al., 2011). In recent years, IMCT has been actively utilized in the intervention and treatment of various conditions, including Alzheimer’s disease, stroke, Parkinson’s disease, diabetes, and cancer. However, the advantages afforded to cognitive and physical functions depend not only on the characteristics of the physical activities but also on the specific cognitive domain and cognitive workload experienced during training.

Cognitive workload influences both the learning process and skill retention among older adults. Evidence suggests that cognitive load can influence cognitive performance in older adults under single-task conditions (Vermeij et al., 2014). In working-memory paradigms, increasing task load is generally associated with poorer behavioral performance, including reduced accuracy and longer reaction times, and these load-related costs become more apparent at higher levels of task difficulty (Isingrini et al., 2015). Further studies indicate that older adults often recruit greater executive-control resources to maintain performance under increasing task demands; however, such compensation may not remain effective as load continues to rise (Vermeij et al., 2014). This underscores the importance of considering cognitive load in the design of training programs for older adults, as excessive cognitive demands may hinder both performance and learning.

Notably, a motor-cognitive training regimen that targets a particular cognitive domain and imposes suitable cognitive demands is likely to provide more significant benefits for both the body and mind (Tao et al., 2022). Despite the potential benefits of IMCT, the effectiveness of exergames in the market is often limited by their inability to dynamically adjust to users’ cognitive and physical needs. Most exergame systems provide a restricted range of scenarios and preset tasks that are not easily adaptable to individual cognitive loads or specifically targeted to address distinct cognitive domains. This lack of flexibility can impede the optimization of training outcomes, as the cognitive challenges presented may not always align with the current capabilities or training objectives of users.

In this study, we utilized the measurement and training system (GlobalSpeed GmbH, Hemsbach, Germany), an interactive training system capable of adjusting cognitive load according to individual differences, to conduct acute IMCT for the participants. This system’s ability to modulate cognitive demands in real-time makes it an ideal tool for investigating the impact of varying cognitive loads on executive functions and postural control. The objective of the present study was to examine the effects of IMCT programs with three levels of cognitive load (high, medium, and low) on executive function, spatio-temporal gait parameters, and postural control in middle-aged and older adults and to further identify which outcome measure best discriminates among different cognitive load conditions. By leveraging the customizable features of SpeedCourt, we aimed to gain a deeper understanding of how different cognitive workloads influence cognitive and motor outcomes, thereby informing the development of more effective fall prevention strategies tailored to individual needs.

## 2 Methods and materials

### 2.1 Study design

The study employed a single-blind, parallel-group randomized controlled trial (RCT) design to examine the acute effects of varying cognitive loads on EF, postural stability, and single/dual-task gait performance in middle-aged and older adults during IMCT. Participants were stratified by age and gender and then randomly assigned to one of three groups using block randomization: the low cognitive load group (LCG), the medium cognitive load group (MCG), or the high cognitive load group (HCG). The motor-cognitive training sessions were conducted on different days, with each participant assigned to a specific cognitive load level (as outlined in the procedure section). Prior to the sessions, participants were instructed to avoid engaging in strenuous physical activity and to refrain from excessive food intake on the day of the experiment. Heart rate was continuously monitored during the exercise sessions to control for the potential influence of exercise intensity on the dependent variables. Outcome assessors who administered the executive-function, gait, and postural-control measurements, as well as the personnel responsible for statistical analysis, were blinded to group assignment; group labels were replaced with anonymised codes prior to analysis. Because of the nature of the IMCT intervention, participants and the trainer who delivered the training session could not be blinded.

### 2.2 Participants

#### 2.2.1 Recruitment

Participants for this study were recruited from the communities surrounding the Shandong Sports Training Center located in Jinan, China, using a convenience sampling approach. An *a priori* power analysis was conducted using G*Power 3.1.9.7. The calculation was based on an F test for repeated-measures ANOVA, within-between interaction, corresponding to the planned comparison of pre-to-post changes among the three groups. The parameters were set as follows: three groups, two repeated measurements, a medium effect size of Cohen’s *f* = 0.25, ɑ = 0.05, and statistical power = 0.80. Under these assumptions, the required total sample size was 42 participants. Accordingly, 42 participants were recruited, with 14 participants in each group, and all participants completed the study and were included in the final analysis.

#### 2.2.2 Inclusion and exclusion criteria

Prior to the commencement of the experiment, potential participants completed the Physical Activity Readiness Questionnaire (PAR-Q) and a health history questionnaire. The researchers subsequently screened these individuals based on specific inclusion and exclusion criteria. The inclusion criteria were as follows: (1) age between 45 and 74 years; (2) normal cognitive function, as assessed by the Montreal Cognitive Assessment (MoCA) with a score of ≥ 26 (Vásquez et al., 2019); (3) no cardiovascular, pulmonary, metabolic, visual, or auditory diseases; (4) no neurological or psychiatric disorders; (5) ability to walk independently for 6 min; and (6) no contraindications to exercise, as determined through PAR-Q screening.

The exclusion criteria included: (1) severe physical illness or health problems, such as severe fractures or bone and joint diseases, that affected gait or balance ability; (2) current participation in other interventions or training that could influence cognition or physical function; (3) severe visual or hearing impairment that could affect the conduct of the experiment or interpretation of the results; and (4) recent use of medications that could affect cognitive or motor performance (e.g., antidepressants or hypnotics).

#### 2.2.3 Ethical considerations

The procedures and methods of this study were approved by the Institutional Review Board of Shandong Sport University to ensure the ethical and safe treatment of human subjects (reference number: 2022041). Informed written consent was obtained from all participants prior to the commencement of the experiment. To ensure participant well-being, individuals were required to rest for at least 15 min on-site after each motor-cognitive training session to address any physical discomfort. If participants reported symptoms such as dizziness or nausea, prompt medical attention would be provided. In cases of exercise-related injuries, such as falls or sprains, the study involving those participants would be terminated. Participants were permitted to leave only after confirming the absence of any adverse symptoms.

### 2.3 Procedure

**Figure 1:**
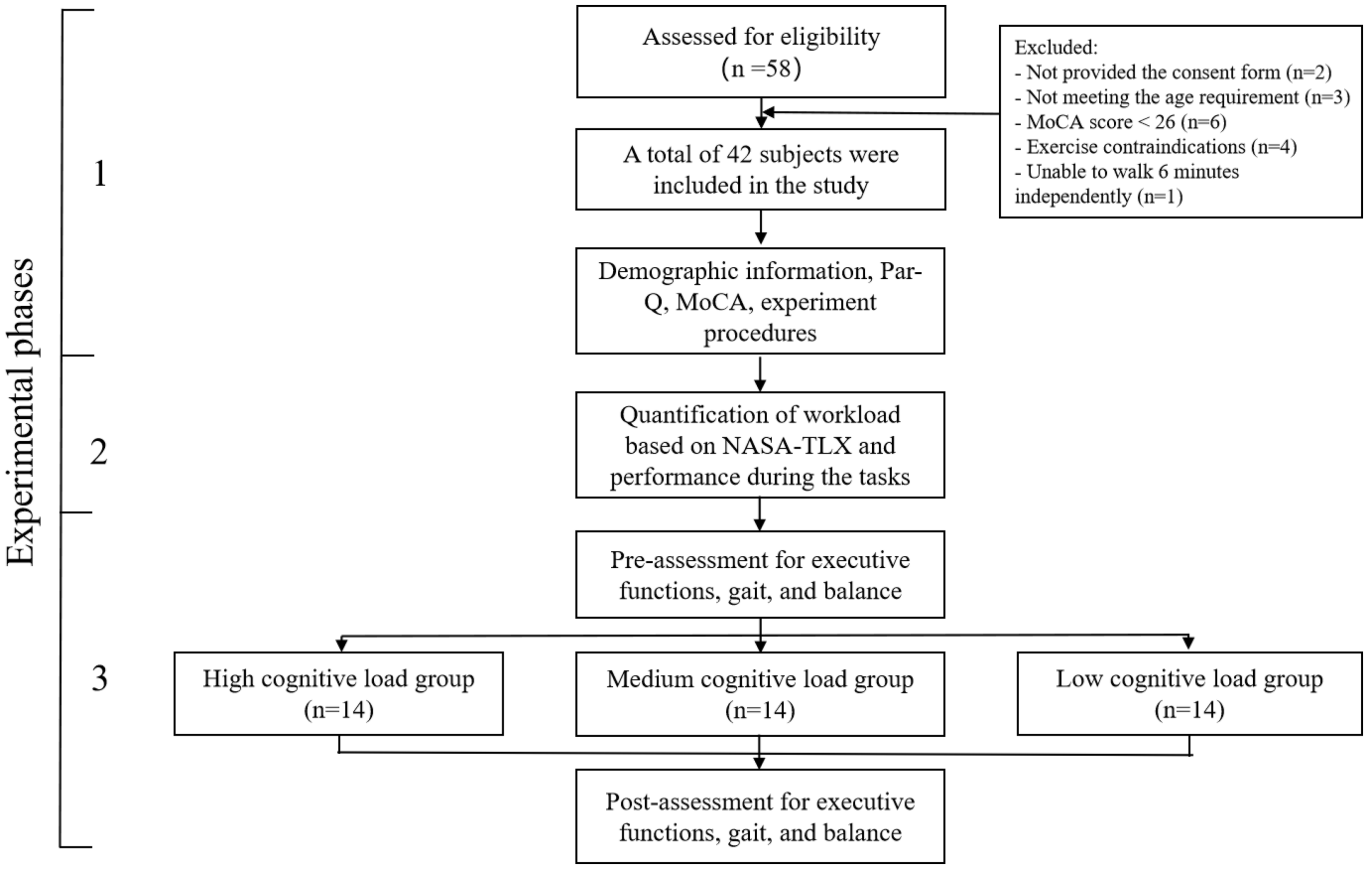
Flow diagram of the procedure of the study.

All participants completed the experiment in three phases (Figure 1). During their initial visit to the experimental site, each participant signed a written informed consent form and completed a demographic questionnaire, the PAR-Q, and the MoCA Scale. The researchers presented the experimental protocol to the participants, which included an overview of cognitive load determination in IMCT, the methodology for IMCT, and the assessment of EF, balance ability, and single/dual-task gait.

During the second visit, the researchers selected motor-cognitive tasks from the “Speed Memory”^1^ and “Speed Brain”^2^ modules of the system to evaluate participants. NASA Task Load Index (NASA-TLX; Hart and Staveland, 1988) was used to assess the cognitive workload of the participants during the motor-cognitive tasks (refer to the Quantification of cognitive workload section). Based on the test results, the tasks were categorized into three cognitive load levels: high, medium, and low.

On their third visit to the experimental site, participants were randomly assigned to IMCT groups with varying cognitive loads (high, medium, and low) and participated in 30 min of IMCT (A 72-h interval was scheduled between the second and third sessions.). During the training, participants wore a Polar M400 heart rate monitor to continuously record their heart rate throughout the entire exercise session, and the average heart rate during exercise was calculated. The 10-point Borg Rating of Perceived Exertion (RPE) scale was used to assess participants’ subjective perception of exercise intensity every 5 min throughout the session. Before and immediately after the training, participants underwent assessments of balance, gait, and EF.

### 2.4 System

**Figure 2:**
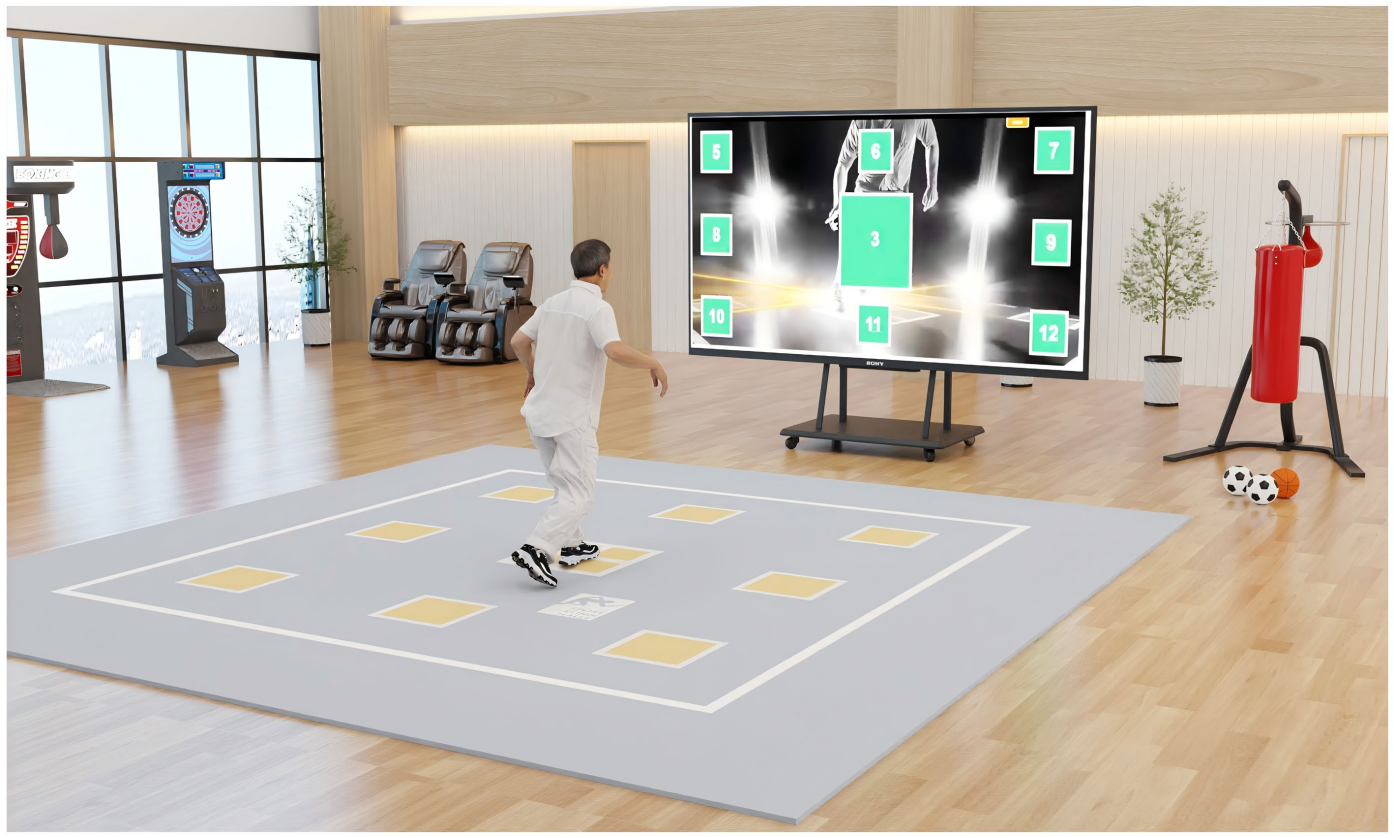
Participants engaging in training using the SpeedCourt system.

The study employed the system (SC650 Professional, Innervations, Germany)–an integrated platform combining a sensor-embedded contact mat, RFID technology, SpeedPro® software, and real-time visual feedback–to deliver IMCT. Participants customized training parameters through the software interface while responding to on-screen cognitive stimuli via foot movements and directional running. Sensor-derived motion data generated instant performance metrics displayed during sessions. Figure 2 depicts the scene of participants engaging in training using the system.

### 2.5 Quantification of cognitive workload

To eliminate subjective assumptions and objectively assess the cognitive load of participants, the study employed a task-based performance quantification method. This approach ensured that the classification of low, medium, and high load levels was based on behavioral responses of the participants rather than subjective judgments by researchers, thereby enhancing the reliability of the results. The “Speed Memory-Classic” and “Speed Brain-Classic” training modules of the system were selected to quantify the cognitive load of participants during IMCT. Three primary reasons justify the selection of these two modules: (1) Compared to modules such as “Jump,” “Speed Chase,” and “Reaction,” which impose relatively high demands for movement speed and lower limb explosive power, the exercise forms and intensities of the “Speed Memory” and “Speed Brain” modules are more appropriate for middle-aged and older adults. (2) Beyond enhancing spatial orientation ability, the training provided by these two modules requires greater cognitive engagement, including working memory and computational skills. (3) These modules offer multiple difficulty levels, ranging from easy to difficult, thereby facilitating the quantification and classification of cognitive load.

The task difficulty of the “Speed Memory - Classic” module was categorized into seven levels, ranging from 1 to 7. At level 1, participants were required to remember a single digit, which was randomly generated by the system from the range of 1 to 12. At level 2, participants had to recall two simultaneously presented digits, also randomly generated. This pattern continued up to level 7. The digit or digit string was displayed on the front screen for 3,000 milliseconds before disappearing, after which participants stepped on the corresponding squares on the floor mat in the correct sequence according to their memory. Similarly, the difficulty of the “Speed Brain-Classic” module was divided into seven levels, involving random addition, subtraction, multiplication, and division operations within the ranges of 10, 25, 50, 100, 250, 500, and 800, respectively. The arithmetic expression was shown on the front screen for 3,000 milliseconds before it disappeared, prompting participants to step on the corresponding squares based on their calculated results. Prior to the formal testing, participants were given the opportunity to familiarize themselves with both tasks and practice. During the formal test, each difficulty level was assessed 10 times.

The NASA-TLX scale and performance were employed to evaluate the cognitive workload associated with IMCT. The NASA-TLX scale divides cognitive load factors into six components: physical demand, mental demand, temporal demand, performance, effort, and stress. Each component is represented by a linear scale divided into 10 equal segments. A higher total load score indicates a greater level of cognitive load. Performance is measured by the number of correct answers per minute provided by participants during the task (DeNisi and Murphy, 2017); thus, a higher number of correctly completed tasks per unit of time correlates with improved performance.

**Figure 3:**
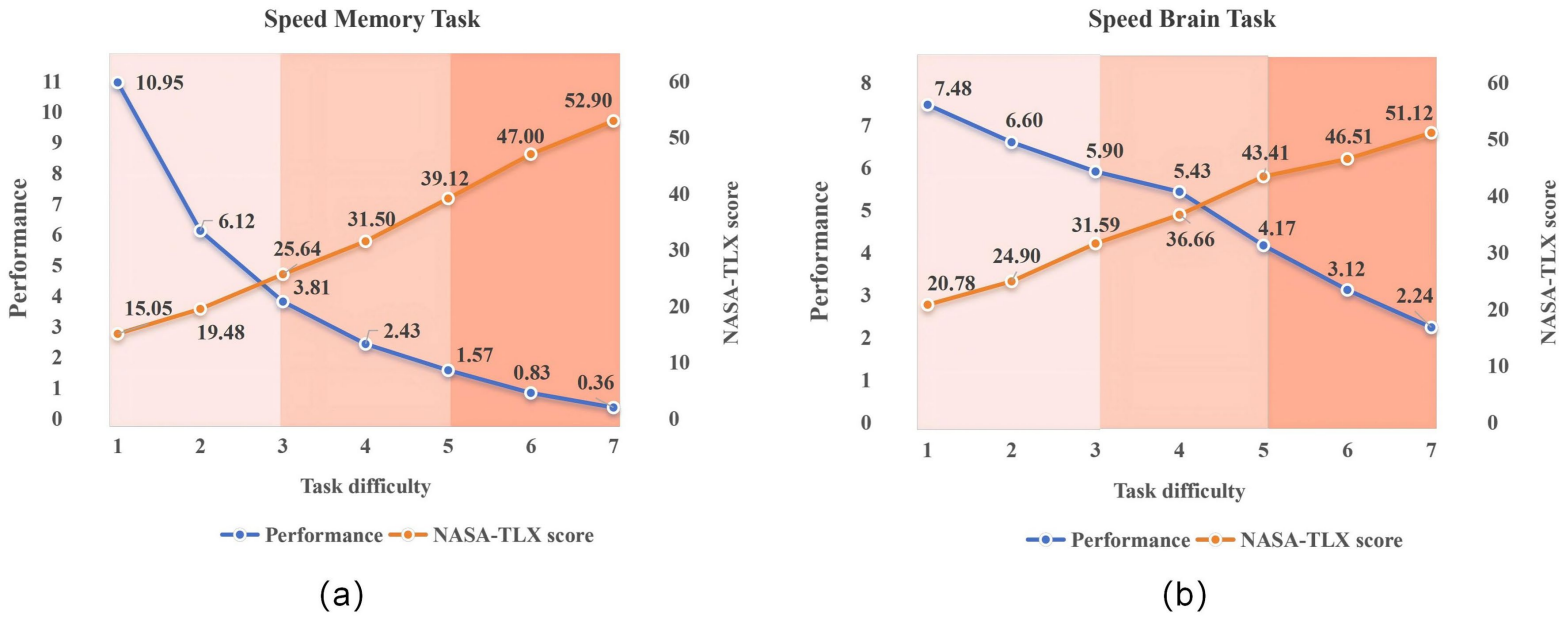
**(a)** Changes in performance (Speed Memory Task) and NASA-TLX scores of participants as task difficulty increases. **(b)** Changes in performance (Speed Brain Task) and NASA-TLX scores of participants as task difficulty increases.

As shown in Figure 3, both NASA-TLX scores and task performance exhibited a progressive trend with increasing task difficulty. At the highest difficulty level (level 7), NASA-TLX scores exceeded 50 points, indicating substantial cognitive workload, while performance dropped markedly to 0.36 (Speed Memory) and 2.24 (Speed Brain), reflecting cognitive overload. These results confirm that the selected tasks effectively induced a range of cognitive loads across difficulty levels.

To operationalize cognitive load levels for the intervention, we adopted an equal-interval division method based on the difficulty scale (1 to 7). This approach ensured consistent and interpretable load levels across groups while maintaining feasibility for experimental design. Specifically, difficulty levels 1–3 were classified as low cognitive load, 3–5 as moderate, and 5–7 as high. For the training session, we selected the intermediate values of each interval–2, 4, and 6–to represent low, moderate, and high cognitive load, respectively. This selection balances distinctiveness between conditions and avoids ceiling or floor effects. This pre-intervention calibration approach is consistent with Cognitive Load Theory, which conceptualizes intrinsic cognitive load as being determined by both task complexity and the learner’s prior knowledge or expertise (Sweller et al., 2019). It is also compatible with the NASA-TLX framework, which is designed to obtain subjective workload estimates during or immediately after task performance (Hart, 2006).

### 2.6 Intervention

**Figure 4:**
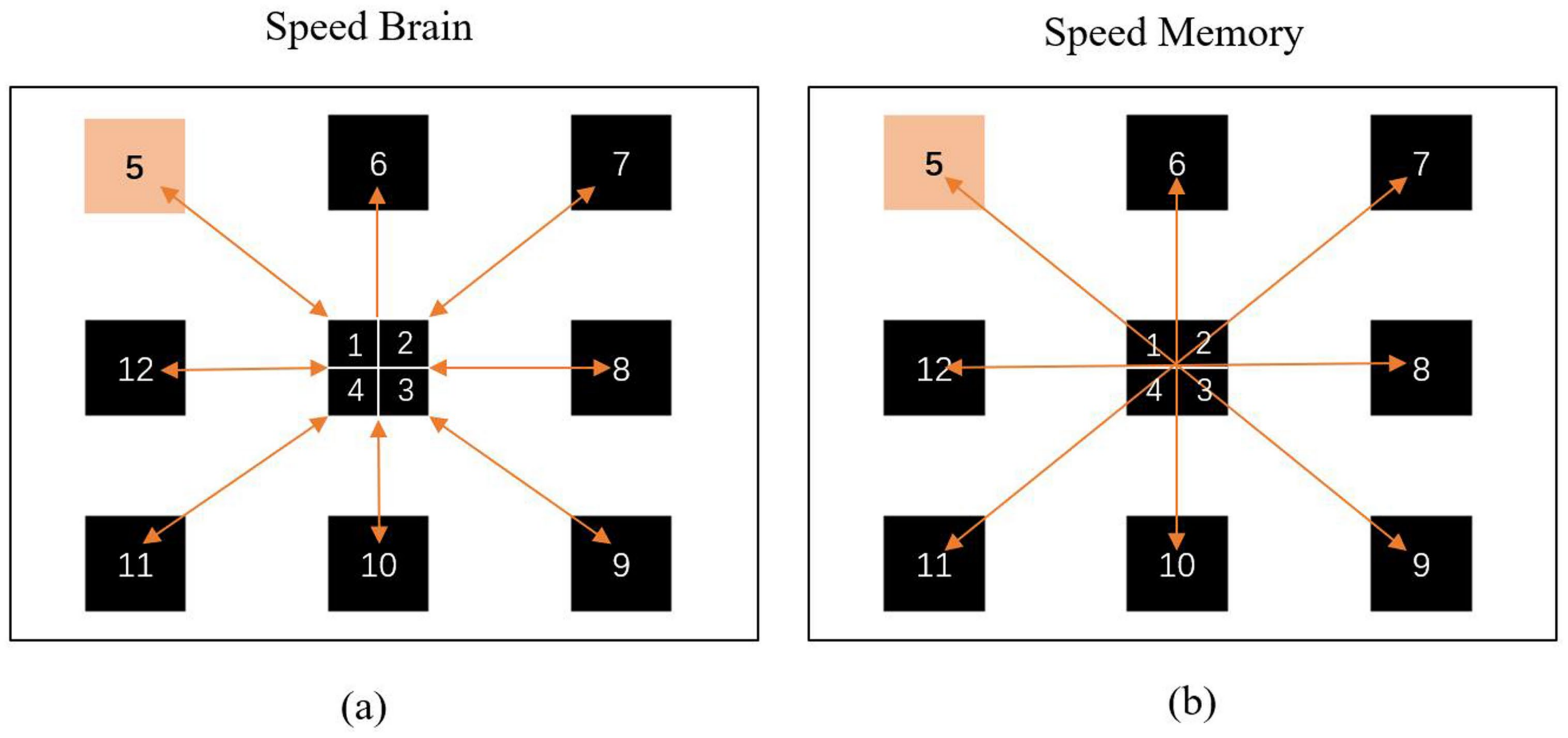
**(a)** Schematic of the Speed Brain mode task. **(b)** Schematic of the Speed Memory mode task.

Participants engaged in a one-time IMCT session using the training system. The total training duration was 30 min, which included 5 min of warm-up exercises, 20 min of IMCT, and 5 min of cooling-down exercise. The “Speed Memory” and “Speed Brain” training modules were used for the participants, with each section lasting 10 min. The three experimental conditions utilized motor-cognitive tasks characterized by high, medium, and low cognitive load, as defined in the previous phase of the experiment. In the “Speed Memory” mode, one or more digital stimuli were displayed on a large screen. The stimuli appeared simultaneously and then quickly disappeared. Participants were required to memorize the numbers displayed on the screen and run to the corresponding number on the designated area (a floor mat equipped with built-in sensors). In the “Speed Brain” mode, stimuli were presented in the form of mathematical calculations, and participants were tasked with completing these calculations by navigating to the corresponding areas within the training field (Figure 4). Each task consisted of 4 to 5 sets of training, with each set lasting 2 min and a 30-s rest period between sets.

### 2.7 Outcome measures

#### 2.7.1 Executive functions

The EF tasks, including the Stroop task, more-odd shifting task, and 2-back task were designed and administered using E-Prime 3.0 software. Prior to the commencement of the tests, participants were seated comfortably in front of a computer, ensuring their eyes were horizontally aligned with the screen, positioned 60 cm away. The participants were briefed on the usage of the reaction keys and the experimental procedure to familiarize themselves. To mitigate the learning effect, participants underwent several blocks of practice trials before the formal assessment. The experiment formally began once the participants achieved an accuracy rate of 80% in the practice sessions, indicating a satisfactory mastery of the testing process.

##### 2.7.1.1 Inhibitory function

**Figure 5:**
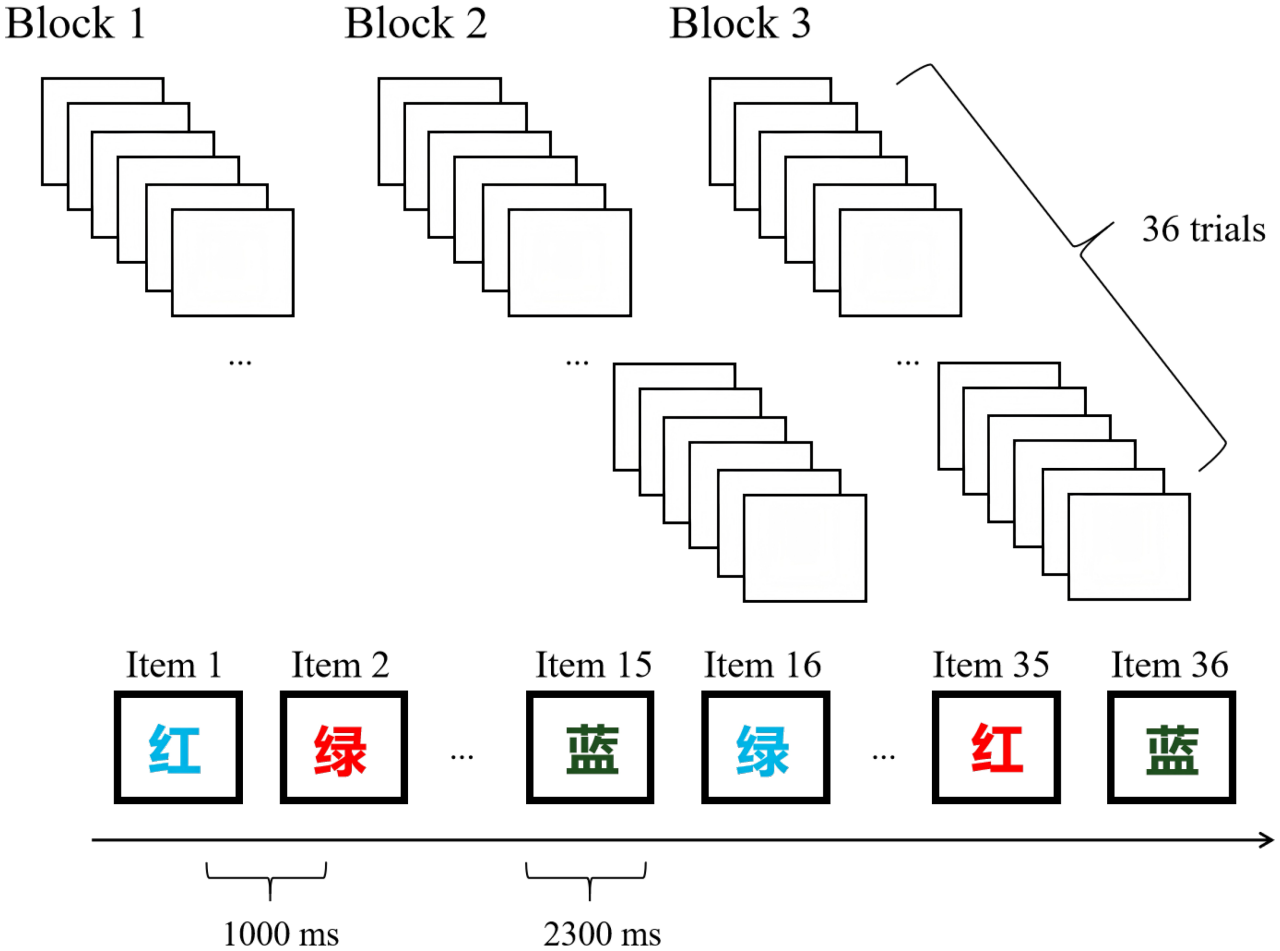
Procedures of the Stroop task.

The Stroop task was adopted to evaluate the inhibitory function of the participants. In the Stroop task, four Chinese characters, namely “红,” “绿,” “黄,” and “蓝,” were displayed in red, green, yellow, or blue on the computer screen. Participants were required to respond to the color of the Chinese characters while inhibiting responses to the meaning of the Chinese characters. The congruent condition refers to the matching of the color and meaning of the Chinese characters, while the incongruent condition occurs when the color and meaning do not match. At the beginning of the experiment, participants fixated on the “+” sign at the center of the computer screen for 1,000 ms, followed by a stimulus presented for 1,500 ms. After the stimulus ended, participants had to respond within 2,300 ms (Figure 5).

Participants were instructed to press “D” key for red, “F” for green, “J” for yellow, and “K” for blue characters. The dependent variables included response accuracy (%), reaction time (ms), and Stroop interference scores. The Stroop accuracy interference score was calculated as the congruent accuracy (%)—the incongruent accuracy (%), while the Stroop reaction time interference score was the average reaction time for incongruent trials (ms)—the reaction time for congruent trials (ms).

##### 2.7.1.2 Cognitive flexibility

**Figure 6:**
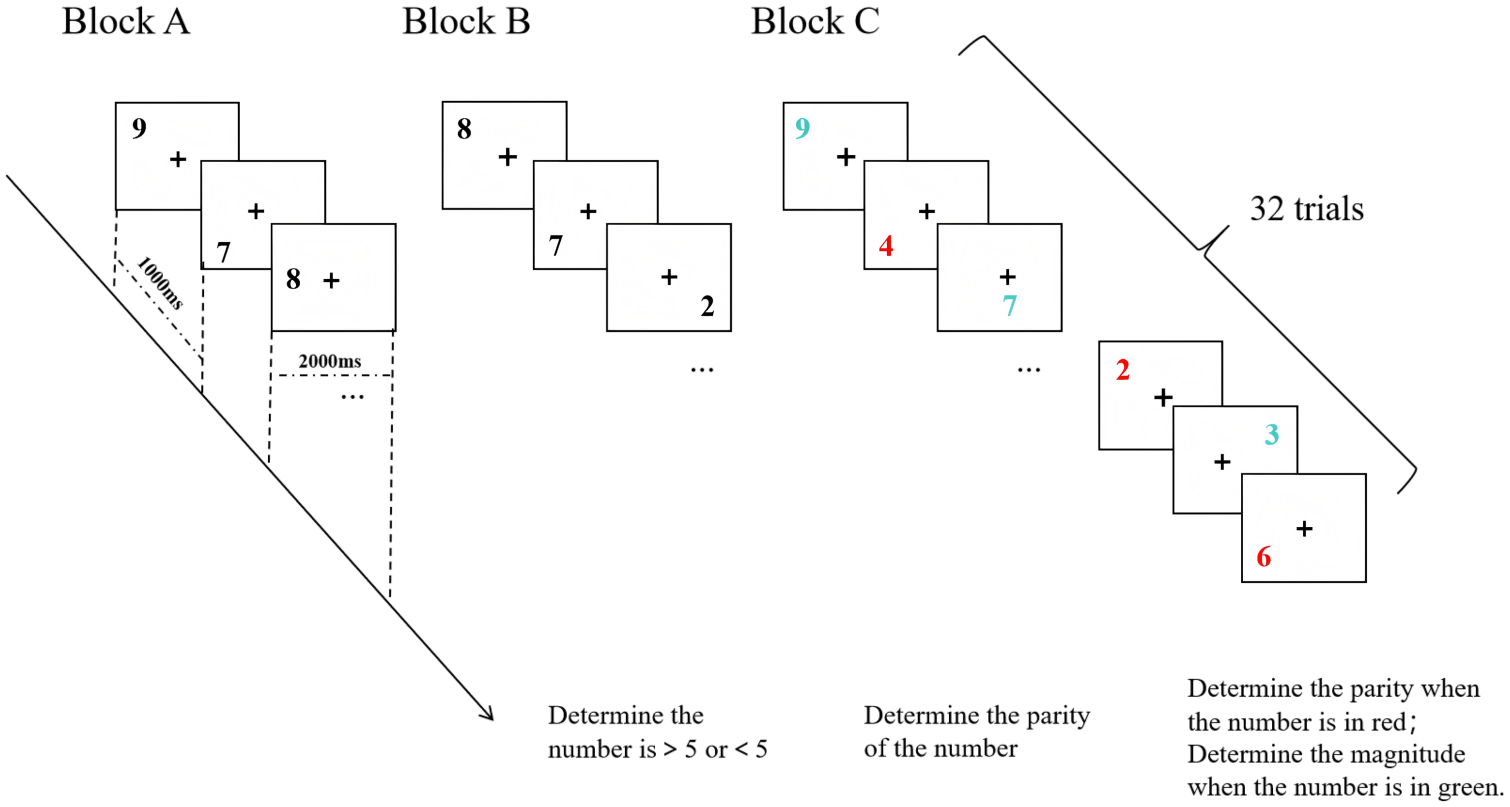
Procedures of the more-odd shifting task.

Cognitive flexibility was evaluated using the more-odd shifting task. The task involved numbers 1 to 9 (excluding 5) being displayed for 2000 ms, with a 1,000 ms interval between each stimulus. The task comprised three types of blocks. Block A consisted of 16 non-switching trials in which participants were tasked with determining whether the number on the screen was greater than or less than 5 by pressing the “F” key for less than 5 and “J” key for greater than 5. In Block B, participants completed 16 non-switching trials where they were required to determine the parity of the number displayed by pressing “F” key for odd and “J” key for even. Block C comprised 32 switching trials in which participants were tasked with determining both the magnitude and the parity of the numbers. When presented with numbers in red, participants were instructed to indicate their on magnitude by pressing “F” for numbers less than 5 and “J” for numbers greater than 5. When presented with numbers in green, participants needed to determine parity by pressing “F” for odd and “J” for even. A total of 6 blocks were tested and balanced according to the ABCCBA order (Figure 6). The mean reaction time and response accuracy (%) for correct responses were recorded for each type of block (switching, non-switching), as well as the switching cost, which was the difference between the mean reaction time of switching blocks and non-switching blocks.

##### 2.7.1.3 Working memory

**Figure 7:**
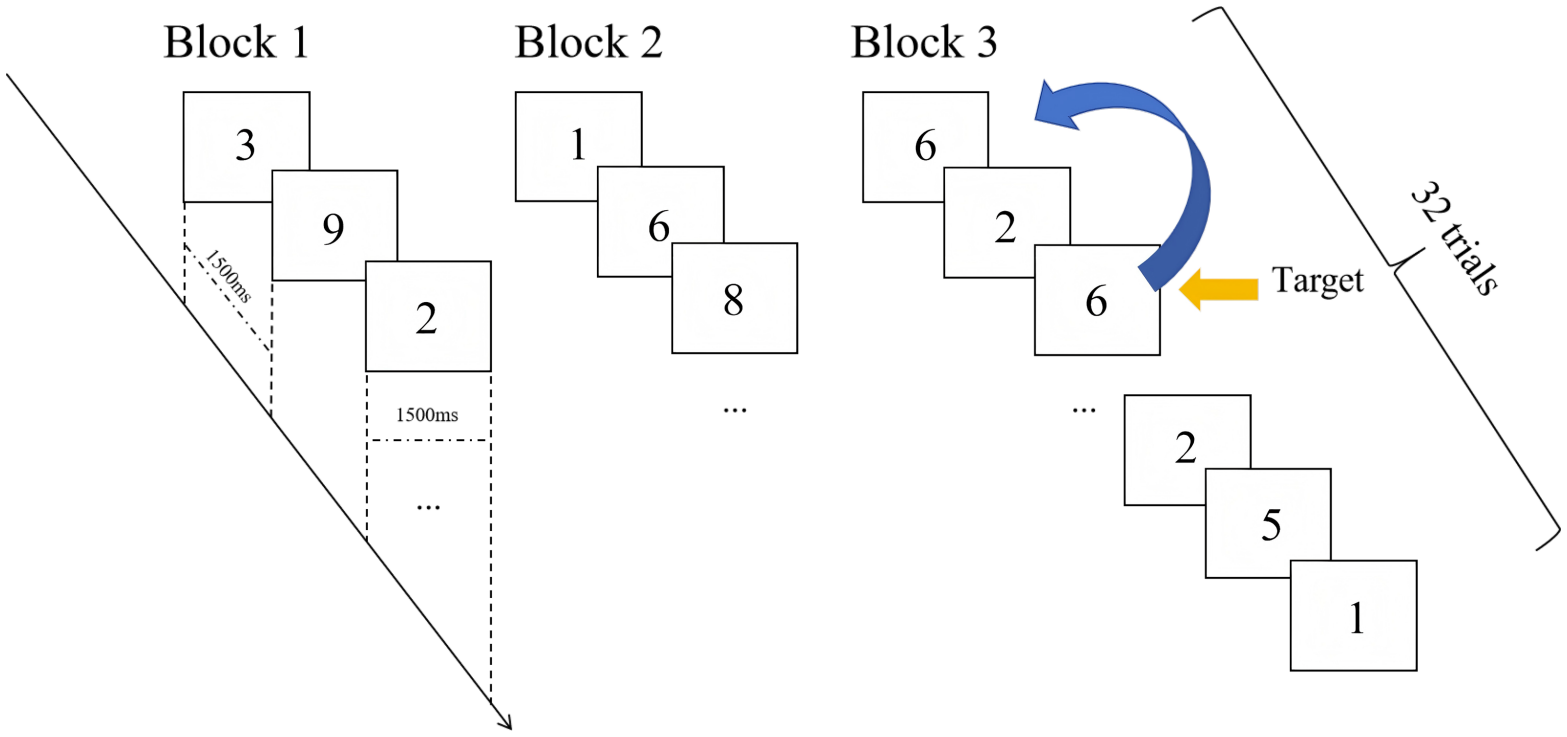
Procedures of the 2-back task.

A numerical 2-back working memory task was used in this study. At the start of each trial, a stimulus (a random digit from 0–9) was presented visually every 3,000 ms (with a 1,500 ms stimulus duration and a 1,500 ms interstimulus interval; Figure 7). If the stimulus displayed matched the one presented two trials earlier, participants were required to press the “F” key; otherwise, they were instructed to press the “J” key. The mean reaction time and response accuracy (%) were recorded.

#### 2.7.2 Gait characteristics

The OptoJump infrared optical testing and analysis system (Microgate, Italy) was employed to assess the gait performance of the participants, sampling at a rate of 1,000 Hz. During the single-task gait test, participants walked at a self-selected speed through a 6-meter walkway under undisturbed conditions. The starting point was set two meters from the front end of the walkway, and participants continued walking until they exceeded the end of the walkway by 2 meters, thus eliminating the influence of acceleration and deceleration during the initial and terminal phases on the results. The dual-task walking test required participants to continuously walk back and forth along the 6-meter walkway for 1 min, maintaining a self-selected speed while simultaneously performing a cognitive task. This cognitive task entailed consecutively subtracting 3 from a randomly assigned three-digit number between 200 and 300, with participants verbally reporting the results.

For each task condition, one practicing trial and two formal trials were conducted. The following spatiotemporal parameters were recorded: gait speed, step length, stride length, double-support time, double-support phase, step length coefficient of variability (CV), propulsion phase CV, and double-support phase CV. Additionally, to comprehensively assess the performance of the participants, cognitive task accuracy and dual-task costs (DTC) were also calculated. Cognitive task accuracy was determined by calculating the proportion of correct responses to the total number of attempts (Howell et al., 2016). The DTC was calculated using the formula: DTC = (dual-task walking speed - single-task walking speed) / single-task walking speed × 100% (Chen and Pei, 2018).

#### 2.7.3 Postural control

Postural control was assessed using the K-FORCE balance force plate (Kinvent, Montpellier, France), featuring high-precision sensors (1,000 Hz sampling) across two 320 mm × 160 mm platforms. Participants performed pre−/post-training evaluations involving two 30-s trials each of double-leg standing with eyes open (DLEO) and eyes closed (DLEC) under standardized conditions: quiet environment, visual fixation on a wall 1 m from the plates. Baseline testing followed 5-min rest periods.

Standardized stance preparation included 30° toe angulation and 5 cm heel separation, arms relaxed at sides. Participants minimized extraneous movement while maintaining gaze direction during assessments. Each condition incorporated practice trials to familiarize users with task requirements and system feedback. Data were analyzed via integrated BioAnalysis software (AMTI, Version 2.2, Watertown, MA), calculating center of pressure (COP) metrics: sway area, mean velocity, and anteroposterior/mediolateral displacement amplitudes.

### 2.8 Statistical analysis

All statistical analyses were conducted using IBM SPSS Statistics version 27.0 (IBM Corp., Armonk, NY, USA). Data are presented as mean ± standard deviation. Normality and homogeneity of variances were assessed using Shapiro–Wilk and Levene’s tests, respectively. One-way ANOVA was applied to determine significant differences in anthropometric and exercise intensity during the IMCT training, among the three cognitive workload groups: low, medium, and high. For each outcome domain (EF, postural control, and gait parameters), pre- to post-intervention change scores (*Δ*) were calculated for all relevant variables. A series of one-way multivariate analyses of variance (MANOVA) were performed with Group (LCG, MCG, HCG) as the independent variable and the change scores of each domain as dependent variables; Pillai’s trace was used as the multivariate test statistic. Following a significant multivariate effect, discriminant function analysis (DFA) was conducted to identify the linear combinations of change scores that best discriminated the groups.

Subsequent one-way ANOVAs were conducted on the individual change scores within each domain. The resulting *p* values were adjusted using the Holm-Bonferroni procedure within each domain. For outcomes that remained significant after Holm correction, post-hoc pairwise comparisons were also adjusted using the Holm procedure. Adjusted p values are reported as p_Holm. Partial eta squared (*η_p_^2^*) was reported for omnibus tests, and Cohen’s d or Hedges’ g was reported where appropriate. To assess whether educational attainment influenced the primary outcomes, sensitivity analyses were conducted using one-way ANCOVAs with educational level entered as a covariate. These analyses were performed separately for each outcome domain (EF, postural control, and gait parameters) to verify the robustness of the primary findings. All statistical tests were two-sided, with *α* set at 0.05.

## 3 Results

### 3.1 Participant characteristics

A total of 58 adults were screened for eligibility. Sixteen were excluded before randomization because they did not meet the inclusion criteria, leaving 42 participants who were randomized equally to the LCG, MCG, and HCG (*n* = 14 per group). All 42 randomized participants completed the study. The anthropometric measurement and exercise-intensity data are presented in Table 1. The results of one-way ANOVAs indicated that there was no significant difference in any of the anthropometric measurements among the LCG, MCG, and HCG groups (*p* > 0.05). Moreover, no statistical differences were detected in heart rate or RPE scores during the exercise session (*p* > 0.05).

**Table 1:** Comparison of anthropometric and exercise intensity measurements (*n* = 42).

Variables	LCG (*n* = 14)	MCG (*n* = 14)	HCG (*n* = 14)	*F*(2,39)	*p*
Age (year)	56.86 ± 9.14	58.86 ± 9.98	58.50 ± 11.04	0.16	0.86
MoCA	27.64 ± 1.82	27.79 ± 1.19	28.00 ± 1.75	0.17	0.84
Height (cm)	164.29 ± 7.47	165.86 ± 8.56	162.93 ± 6.49	0.53	0.59
Weight (kg)	66.39 ± 9.52	70.43 ± 9.32	64.04 ± 7.12	1.92	0.16
BMI	24.55 ± 2.69	25.55 ± 2.18	24.07 ± 1.55	1.66	0.20
Heart rate (bpm)	116.29 ± 9.85	116.21 ± 9.43	109.07 ± 13.08	2.02	0.15
RPE	4.73 ± 0.82	4.57 ± 0.67	4.07 ± 0.85	2.73	0.08
Education (year)	12.14 ± 3.21	12.29 ± 3.75	12.50 ± 3.82	0.03	0.966

Data are presented as Mean ± SD. LCG, low cognitive load group; MCG, medium cognitive load group; HCG, high cognitive load group; MoCA, montreal cognitive assessment; RPE, Rating of Perceived Exertion.

All 42 participants completed the intervention and both the pre- and post-assessments, with no loss to follow-up after randomization. No adverse events (e.g., falls, dizziness, sprains, or exercise-related discomfort requiring medical attention) were reported during the training or assessment sessions.

### 3.2 Executive functions

The analysis revealed a statistically significant multivariate main effect for cognitive load group on the composite of EF change scores, Pillai V = 0.804, *F*(20, 62) = 2.086, *p* = 0.015, *η_p_^2^* = 0.402, indicating that the pattern of cognitive changes differed significantly across the LCG, MCG, and HCG (see Table 2 for descriptive statistics).

**Table 2:** Pre and posttest changes in executive functions (*n* = 42).

Executive function	Task conditions	LCG (*n* = 14)		MCG (*n* = 14)		HCG (*n* = 14)		One-way ANOVA on Δ change scores				MANOVA on Δ scores		
		Pre	Post	Pre	Post	Pre	Post	*F*(2,39)	*p*	*p_* Holm	*η_p_* ^2^	*F*(20,62)	*p*	*η_p_* ^2^
Stroop task														
Correct rate (%)	Incongruent	86 ± 12	94 ± 4	87 ± 8	98 ± 3	83 ± 13	90 ± 6	0.52	0.598	>0.999	0.026	-	-	-
	Neutral	95 ± 4	98 ± 4	95 ± 6	98 ± 4	94 ± 8	98 ± 3	0.24	0.788	>0.999	0.012	-	-	-
Reaction time (ms)	Incongruent	1,325 ± 249	1,150 ± 347	1,355 ± 249	1,106 ± 279	1,264 ± 326	1,114 ± 279	1.48	0.240	>0.999	0.071	-	-	-
	Neutral	1,165 ± 298	955 ± 250	1,187 ± 254	774 ± 134	1,238 ± 273	1,151 ± 273	19.61	<0.001	<0.001	0.501	-	-	-
More-odd shifting														
Correct rate (%)	Non-switching	91 ± 7	93 ± 6	93 ± 9	95 ± 4	92 ± 7	95 ± 5	0.03	0.975	>0.999	0.001	-	-	-
	Switching	84 ± 11	90 ± 11	87 ± 10	93 ± 10	84 ± 17	88 ± 13	0.34	0.711	>0.999	0.017	-	-	-
Reaction time (ms)	Non-switching	881 ± 199	773 ± 149	882 ± 142	789 ± 145	846 ± 153	800 ± 131	2.15	0.130	>0.999	0.099	-	-	-
	Switching	1,062 ± 239	980 ± 182	1,095 ± 140	968 ± 185	1,094 ± 157	1,006 ± 194	0.51	0.606	>0.999	0.025	-	-	-
2-back														
Correct rate (%)		57 ± 19	72 ± 25	63 ± 19	62 ± 24	68 ± 17	65 ± 24	3.32	0.047	0.419	0.146	-	-	-
Reaction time (ms)		1,019 ± 224	1,037 ± 213	1,137 ± 217	1,061 ± 181	1,124 ± 136	1,059 ± 205	0.63	0.538	>0.999	0.031	-	-	-
MANOVA (overall effect)												V = 0.804	0.015	0.402

Data are presented as mean ± SD. LCG, low cognitive load group; MCG, medium cognitive load group; HCG, high cognitive load group. p_Holm refers to Holm-adjusted p values for follow-up one-way ANOVAs on change scores within the EF domain. Detailed Δ values, paired 95% CIs, and percentage changes are reported in Supplementary Table S1.

To further explore how the three groups were discriminated by the cognitive changes, a DFA was performed. Two discriminant functions were extracted. Function 1 explained 83.6% of the between-group variance (canonical R^2^ = 0.766), whereas Function 2 explained the remaining 16.4% (canonical R^2^ = 0.467). According to the structure matrix, the change in Stroop neutral reaction time *Δ* loaded most strongly on Function 1 (*r* = 0.84), whereas the change in 2-back accuracy Δ loaded most strongly on Function 2 (*r* = 0.78). Classification analysis showed that 78.6% of the original cases were correctly classified, and 54.8% were correctly classified after cross-validation, indicating moderate discriminative performance. The group centroids plot indicated that Function 1 clearly separated the MCG from the LCG and HCG, while Function 2 distinguished the LCG from the HCG (Supplementary Figure S1).

Follow-up one-way ANOVAs on EF change scores were adjusted using the Holm procedure across the 10 EF outcomes. Only Stroop neutral reaction time remained significant after correction, *F*(2, 39) = 19.612, *p*_Holm < 0.001, *η_p_^2^* = 0.501. The MCG showed the largest reduction in reaction time (*Δ* = −413.6 ms, 95% CI [−507.1, −320.0]; −34.8%), significantly exceeding the reductions in both the LCG (Δ = −209.3 ms, 95% CI [−302.1, −116.5]; −18.0%; *p*_Holm < 0.001, Cohen’s d = 1.27) and the HCG (Δ = −86.9 ms, 95% CI [−132.4, −41.5]; −7.0%; *p*_Holm < 0.001, Cohen’s d = 2.56). The LCG also showed a greater reduction than the HCG (*p*_Holm = 0.026, Cohen’s d = 0.97).

The nominal group effect for 2-back accuracy did not remain significant after Holm correction, *F*(2, 39) = 3.322, *p* = 0.047, *p*_Holm = 0.419,*η_p_^2^* = 0.146. No other EF change scores showed significant corrected group effects (all *p*_Holm > 0.05; Table 2 and Supplementary Table S1).

### 3.3 Gait performance

#### 3.3.1 Dual-task gait performance

The multivariate analysis showed a significant main effect of cognitive load group on dual-task gait outcomes, Pillai V = 1.201, *F*(26, 56) = 3.241, *p* < 0.001,*η_p_^2^* = 0.601, indicating overall differences among the LCG, MCG, and HCG (Table 3).

**Table 3:** Pre and posttest changes in gait performance during the dual-task walking test (*n* = 42).

Spatio-temporal gait parameters	LCG (*n* = 14)		MCG (*n* = 14)		HCG (*n* = 14)		One-way ANOVA on Δ change scores				MANOVA on Δ scores		
	Pre	Post	Pre	Post	Pre	Post	*F*(2,39)	*p*	*p_*Holm	*η_p_* ^2^	*F*(26,56)	*p*	*η_p_* ^2^
Gait velocity (m/s)	0.7 ± 0.2	0.8 ± 0.2	0.7 ± 0.2	1.0 ± 0.2	0.7 ± 0.2	0.8 ± 0.2	8.444	0.001	0.012	0.302	-	-	-
Step length (cm)	56 ± 6	56 ± 7	51 ± 6	53 ± 7	54 ± 5	53 ± 6	0.839	0.438	>0.999	0.042	-	-	-
Stride length (cm)	111 ± 12	112 ± 15	103 ± 13	105 ± 15	107 ± 12	107 ± 13	0.176	0.839	>0.999	0.009	-	-	-
Propulsion phase (%)	31 ± 4	30 ± 4	33 ± 8	31 ± 8	31 ± 7	31 ± 8	0.228	0.798	>0.999	0.012	-	-	-
Double support time (s)	0.5 ± 0.1	0.5 ± 0.1	0.6 ± 0.2	0.4 ± 0.1	0.6 ± 0.3	0.6 ± 0.3	6.372	0.004	0.040	0.246	-	-	-
Double support phase (%)	60 ± 5	60 ± 5	58 ± 7	60 ± 8	60 ± 6	60 ± 8	0.172	0.843	>0.999	0.009	-	-	-
Step length CV (%)	22 ± 4	21 ± 7	21 ± 6	21 ± 6	20 ± 7	20 ± 5	0.104	0.902	>0.999	0.005	-	-	-
Propulsion phase CV (%)	108 ± 60	100 ± 31	87 ± 37	74 ± 25	89 ± 34	88 ± 33	0.218	0.805	>0.999	0.011	-	-	-
Double support CV (%)	39 ± 28	35 ± 25	34 ± 25	26 ± 15	41 ± 24	31 ± 16	0.293	0.748	>0.999	0.015	-	-	-
Attempts	21 ± 8	25 ± 10	25 ± 12	26 ± 13	28 ± 10	26 ± 9	6.193	0.005	0.042	0.241	-	-	-
Correct response	20 ± 9	24 ± 11	24 ± 12	24 ± 14	26 ± 10	24 ± 10	8.355	0.001	0.012	0.300	-	-	-
Dual-task cost (%)	−42 ± 15	−36 ± 15	−42 ± 15	−21 ± 13	−43 ± 18	−34 ± 16	6.524	0.004	0.040	0.251	-	-	-
Cognitive accuracy (%)	91 ± 12	95 ± 6	95 ± 7	90 ± 13	93 ± 9	90 ± 13	2.053	0.142	>0.999	0.095	-	-	-
MANOVA (overall effect)											V = 1.201	0.001	0.601

Data are presented as mean ± SD. LCG, low cognitive load group; MCG, medium cognitive load group; HCG, high cognitive load group. p_Holm refers to Holm-adjusted p values for follow-up one-way ANOVAs on change scores within the dual-task gait/cognitive-performance domain. Detailed Δ values, paired 95% CIs, and percentage changes are reported in Supplementary Table S2.

DFA was conducted, and two discriminant functions were derived. Function 1 explained 52.8% of the between-group variance (canonical R^2^ = 0.784), whereas Function 2 explained the remaining 47.2% (canonical R^2^ = 0.766). Classification analysis showed that 90.5% of the original cases were correctly classified, and 64.3% were correctly classified after cross-validation, indicating good discriminative performance with acceptable stability. The group centroids plot indicated that Function 1 clearly separated the MCG from the LCG and HCG, while Function 2 distinguished the LCG from the HCG (Supplementary Figure S2).

Follow-up one-way ANOVAs on dual-task gait and dual-task cognitive change scores were adjusted using the Holm procedure across 13 outcomes. Significant corrected group effects were observed for gait velocity, double-support time, calculation attempts, correct responses, and dual-task cost (Table 3). For dual-task gait velocity, the corrected group effect was significant, *F*(2, 39) = 8.444, *p*_Holm = 0.012, *η_p_^2^* = 0.302. The MCG showed the largest increase (*Δ* = +0.260 m/s, 95% CI [+0.169, +0.351]; +36.3%), significantly exceeding both the LCG (Δ = +0.086 m/s, 95% CI [+0.048, +0.123]; +12.0%; *p*_Holm < 0.001, Cohen’s d = 1.44) and the HCG (*Δ* = +0.134 m/s, 95% CI [+0.073, +0.195]; +19.6%; *p*_Holm = 0.013, Cohen’s d = 0.94). The LCG and HCG did not differ significantly (*p*_Holm = 0.274).

For dual-task double-support time, the corrected group effect was significant, *F*(2, 39) = 6.372, *p*_Holm = 0.040,*η_p_^2^* = 0.246. The MCG showed a marked reduction (Δ = −0.138 s, 95% CI [−0.214, −0.062]; −24.4%), which was significantly greater than the change observed in the LCG (*Δ* = +0.030 s, 95% CI [−0.036, +0.097]; +6.5%; *p*_Holm = 0.003, Cohen’s d = 1.36). The MCG–HCG and LCG–HCG contrasts were not significant after correction (*p*_Holm = 0.112 and 0.119, respectively).

For dual-task cognitive performance, corrected group effects were significant for calculation attempts, *F*(2, 39) = 6.193, *p*_Holm = 0.042, *η_p_^2^* = 0.241, and correct responses, F(2, 39) = 8.355, *p*_Holm = 0.012, *η_p_^2^* = 0.300. For calculation attempts, the LCG increased attempts (*Δ* = +3.64, 95% CI [+0.272, +7.01]; +17.0%), whereas the HCG showed a descriptive decrease (Δ = −2.07, 95% CI [−4.34, +0.199]; −7.4%); the LCG–HCG contrast was significant (*p*_Holm = 0.004, Cohen’s d = 1.15). For correct responses, the LCG showed the largest increase (Δ = +4.14, 95% CI [+0.870, +7.42]; +20.8%), significantly exceeding both the MCG (*Δ* = +0.500, 95% CI [−1.14, +2.14]; +2.1%; *p*_Holm = 0.047, Cohen’s d = 0.81) and the HCG (*Δ* = −2.14, 95% CI [−3.95, −0.333]; −8.2%; *p*_Holm < 0.001, Cohen’s d = 1.37). The remaining pairwise contrasts for these cognitive outcomes were not significant after correction.

For dual-task cost, the corrected group effect was significant, *F*(2, 39) = 6.524, *p*_Holm = 0.040,*η_p_^2^* = 0.251. The MCG showed the largest shift toward zero, indicating a reduced magnitude of dual-task cost (*Δ* = +20.0 percentage points, 95% CI [+11.5, +28.6]; a 48.6% reduction in DTC magnitude), significantly exceeding both the LCG (Δ = +5.8 percentage points, 95% CI [+2.3, +9.3]; a 13.8% reduction; *p*_Holm = 0.005, Cohen’s d = 1.26) and the HCG (Δ = +8.5 percentage points, 95% CI [+2.4, +14.6]; a 19.9% reduction; *p*_Holm = 0.018, Cohen’s d = 0.89). The LCG and HCG did not differ significantly (*p*_Holm = 0.518).

Other dual-task outcomes, including step length, stride length, propulsion phase, double-support phase, gait variability indices, and cognitive accuracy, did not show significant corrected group effects (all *p*_Holm > 0.05; Table 3 and Supplementary Table S2).

#### 3.3.2 Single-task gait performance

The multivariate analysis showed a significant main effect of cognitive load group on single-task gait outcomes, Pillai V = 0.962, *F*(18,62) = 3.294, *p* < 0.001,*η_p_^2^* = 0.481, indicating overall differences among the LCG, MCG, and HCG.

DFA was conducted, and two discriminant functions were derived. Function 1 explained 52.8% of the between-group variance (canonical R^2^ = 0.458), whereas Function 2 explained the remaining 47.2% (canonical R^2^ = 0.274). Classification analysis showed that 57.1% of the original cases were correctly classified, and 40.5% were correctly classified after cross-validation.

However, after Holm correction across the nine single-task gait outcomes, no univariate group effect survived correction (all *p*_Holm > 0.05; Supplementary Table S3).

### 3.4 Postural control

The multivariate analysis showed no significant main effect of cognitive load group on postural control outcomes, Pillai V = 0.543, *F*(20, 62) = 1.155, *p* = 0.323,*η_p_^2^* = 0.271, indicating no overall differences among the LCG, MCG, and HCG. Accordingly, univariate postural-control results were not interpreted as confirmatory; none survived Holm correction (all *p*_Holm > 0.05; Supplementary Table S4).

### 3.5 Sensitivity analysis

To assess whether educational attainment influenced the primary outcomes, sensitivity analyses were conducted using one-way ANCOVAs with educational level entered as a covariate, performed separately for each outcome domain (Supplementary Table S5). Across all domains, every group effect that was significant in the primary analyses remained significant after adjustment for educational attainment, and educational attainment itself was not a significant covariate for any outcome (all *p* > 0.05).

In the executive function domain, the group effect for Stroop neutral reaction time remained significant after controlling for educational attainment, *F*(2, 38) = 19.857, *p* < 0.001, whereas the covariate was non-significant, *F*(1, 38) = 1.842, *p* = 0.183. In the dual-task gait domain, all five outcomes that were significant in the primary analyses remained significant after adjustment: dual-task gait velocity, F(2, 38) = 8.321, *p* = 0.001; double-support time, F(2, 38) = 6.376, *p* = 0.004; calculation attempts, F(2, 38) = 6.521, *p* = 0.004; correct responses, F(2, 38) = 8.705, *p* < 0.001; and dual-task cost, F(2, 38) = 6.524, *p* = 0.004; educational attainment was not a significant covariate for any of these outcomes (all *p* > 0.05). In the single-task gait and postural control domains, no univariate group effect reached significance in the primary analyses, and educational attainment did not significantly influence any change score in these domains (all *p* > 0.05). Taken together, these sensitivity analyses confirm that the observed intervention effects were robust to differences in participant educational background.

## 4 Discussion

The relationship between cognitive load during dual-task training and its effects on physical and mental functions has been a subject of interest in recent studies. To our knowledge, this study is the first to investigate the effects of IMCT under varying cognitive loads on EF, gait, and postural control in middle-aged and older adults. The findings indicate that IMCT with medium and low cognitive loads produces more favorable outcomes in terms of EF and dual-task walking performance. Our results underscore the importance of incorporating cognitive load considerations into the design of IMCT programs for middle-aged and older individuals.

### 4.1 The impact of IMCT on executive function in middle-aged and older adults

In the current investigation, the Stroop color-word test, 2-back test, and more-odd shifting test were employed to comprehensively assess the EF of the participants. An improvement in task accuracy or a reduction in reaction time is likely to reflect enhancements in the brain’s cognitive control mechanisms, information processing and storage capacities, as well as its proficiency in task-switching operations (Politakis et al., 2022). Numerous previous studies have demonstrated that a single bout of aerobic exercise can acutely improve the EF in older adults (Ishihara et al., 2021; Netz et al., 2023; Pontifex et al., 2019). Previous studies have proposed that such effects may be associated with increased neural activation, general physiological arousal (Perrot and Maillot, 2023), and changes in brain-derived neurotrophic factors such as BDNF (Notaras and van den Buuse, 2019). However, these physiological variables were not measured in the present study. A recent study compared the acute effects of two distinct types of motor-cognitive training modalities on the EF of older adults (Li et al., 2024), the results revealed that both traditional motor-cognitive dual- task training (treadmill exercise+cognitive training), and IMCT (exergame) could significantly enhance the cognitive flexibility of older adults. Moreover, the older adults in the IMCT group exhibited significantly superior inhibitory function compared to those in the treadmill + cognitive training group. Inhibitory-related outcomes are an important aspect of EF and may be sensitive to IMCT (Dhir et al., 2021). During the IMCT session, participants were required to rapidly identify visual and spatial stimuli, process task-relevant information, and generate timely motor responses. The reduction in Stroop neutral reaction time may therefore be better interpreted as an improvement in general information-processing speed and response readiness rather than as evidence of enhanced inhibitory control. This interpretation is consistent with previous work suggesting that motor-cognitive training may acutely engage attentional and cognitive-motor integration processes(Li et al., 2024). However, because no biomarker, neuroimaging, or neurophysiological measures were collected, the underlying mechanisms cannot be determined from the present data.

Interestingly, the improvement in EF was not homogenous but rather influenced by the magnitude of cognitive workload. In this study, the reduction in Stroop neutral reaction time was greatest in the MCG group and was significantly greater than that observed in the LCG and HCG groups. In the context of the 2-back task, participants in the LCG group showed a descriptive within-group improvement in 2-back accuracy following the training intervention, but the group effect did not remain significant after Holm correction. Conversely, there were no substantial within-group changes in the MCG and HCG groups before and after the training. The DFA findings further reinforced this interpretation. Variations in EF across different cognitive load conditions were captured by a limited set of key indicators, rather than being uniformly manifested across all cognitive tests. Among these, Stroop neutral reaction time appeared to be the most sensitive measure for discriminating cognitive load levels, while 2-back accuracy functioned as a secondary descriptive index. The findings align with existing perspectives on cognitive load thresholds. Baldwin (2012) posited that as the cognitive demands of a specific task increase, the neural resources available for secondary tasks are proportionally reduced. When the cognitive workload remains within an individual’s manageable threshold, these tasks can effectively contribute to enhancing an individual’s cognitive performance (Ghani et al., 2020). For example, Barcelos et al. (2015) compared the effects of cycling with a low-cognitive-demand task (bike tour), and a high-cognitive-demand task (video game), on the EF of older adults. The authors reported that, following acute training, the EF, as measured by the Color Trails Test, of the video game group was significantly better than that of the bike tour group. However, when the cognitive workload exceeds an individual’s capacity or when the task demands extensive and complex cognitive control, this beneficial effect may be attenuated or even completely negated (Baldwin, 2012). For instance, Kamijo and Abe (2019) found that when individuals engaged in a cognitively demanding task during aerobic exercise, specifically cycling, the participants exhibited greater individual variability in their performance on the working memory task. Although cycling can have a positive impact on physical health, the combined effect of physical exertion and cognitive demands may lead to cognitive fatigue, thereby negatively affecting working memory and overall cognitive performance.

The findings of the present study partially support Baldwin's (2012) hypothesis, suggesting that maintaining an appropriate level of cognitive workload during motor-cognitive training may be conducive to enhancing selected aspects of task performance. Conversely, an excessive cognitive workload may attenuate these short-term benefits. This finding further underscores the importance of carefully considering the cognitive demands of tasks when designing intervention programs that integrate exercise and cognitive training, with the aim of maximizing the positive impact of exercise on cognitive function (Barcelos et al., 2015). However, because participants completed identical Stroop, 2-back, and more-odd shifting tasks before and after a single intervention session within approximately 1 h, practice effects cannot be ruled out as a contributor to the observed pre–post improvements, and the absence of a non-intervention control group precludes a direct estimate of their magnitude.

It is important to note that there were no significant corrected between-group effects for more-odd shifting task performance after the exercise intervention. According to Kelly et al. (2014), targeted cognitive training can effectively strengthen specific cognitive domains, while general cognitive training may not be sufficient to activate specific cognitive functions. The training tasks employed in this study were predominantly focused on working memory, spatial orientation, and logical reasoning, lacking specific and effective stimuli for cognitive flexibility. Therefore, it is plausible that this may have limited the ability to significantly enhance the participants’ cognitive flexibility performance. Future research should consider incorporating a more diverse range of cognitive tasks to comprehensively promote the improvement of cognitive function across multiple domains.

### 4.2 The impact of IMCT on gait in middle-aged and older adults

Dual-task walking performance has been recognized by numerous studies as a key indicator for assessing the health status of the elderly (Piche et al., 2023). Among these indicators, gait speed serves not only as an important basis for measuring functional decline in older adults but also provides a more accurate reflection of their cognitive and motor function status through the dual-task walking test (Nedović et al., 2024). This is of significant relevance for patients with cognitive impairment, frail older adults, and specific groups at risk of falling. In this study, IMCT with three different cognitive loads were utilized and the results demonstrated that these interventions were associated with improved walking speed during dual-task walking. This finding aligns with previous IMCT interventions. Notably, an important finding of this study is that after engaging in IMCT with moderate cognitive load, participants exhibited a significant improvement in dual-task walking speed compared with the LCG and HCG. They also showed a reduction in dual-task double-support time, with the corrected pairwise contrast reaching significance versus the LCG. In addition, the LCG demonstrated a greater increase in correct responses than the HCG. Furthermore, DFA indicated that dual-task gait velocity was the primary variable distinguishing different cognitive load conditions, whereas the number of correct responses served as a secondary complementary indicator. The classification accuracy of the original cases remained high, suggesting good discriminative performance of the model. Collectively, these findings indicate that dual-task gait is highly sensitive to variations in cognitive load.

In dual-task walking scenarios, such as performing addition and subtraction while walking, improvements in gait speed may indicate positive changes across multiple cognitive abilities. For instance, this could suggest that individuals have enhanced capabilities in allocating and coordinating cognitive resources (Protzak and Gramann, 2021). In a dual-task context, individuals must simultaneously manage both walking and cognitive tasks, necessitating efficient allocation of cognitive resources to ensure that performance in both areas is maintained or even improved (Schlegel et al., 2025). An increase in pace indicates that individuals can better sustain or enhance their walking efficiency while successfully completing cognitive tasks, thereby demonstrating effective allocation and coordination of cognitive resources. Previous studies indicate that, compared to high cognitive load training, lower cognitive load training has a relatively limited impact on improving dual-task pace. This may be attributed to the fact that low cognitive load does not sufficiently challenge the cognitive resources necessary for achieving effective dual-task gait performance, resulting in smaller improvements in gait speed under dual-task conditions (Chen et al., 2024). Conversely, high cognitive load training is likely to produce more pronounced gait deviations and instability. When individuals are required to manage more complex cognitive tasks while walking, they may experience increased variability in stride time and other gait irregularities, with this instability specifically manifesting as variations in stride length and cadence. These parameters are critical for maintaining balance and preventing falls (Meester et al., 2019). In the present study, however, gait variability indices did not show significant corrected group effects, so this mechanism remains inferential.

Secondly, relevant studies have demonstrated a significant correlation between dual-task walking speed and the EFs of the elderly (van Iersel et al., 2008; Watson et al., 2010). An increase in walking speed may be related to EF, particularly cognitive abilities such as planning, organizing, interference control, working memory, and flexible switching (Martin et al., 2013). Furthermore, short-term aerobic exercise and high-intensity endurance training have been proposed to influence EF through neurotransmitter levels, blood flow, and oxygen supply to the brain (Babaei and Azari, 2022; Ludyga et al., 2016; Ye et al., 2024). These physiological pathways were not measured here. Additionally, the experimental results of Li et al. (2024) indicated that following acute IMCT, improvements in inhibitory function were accompanied by a significant enhancement in dual-task speed. The present data are consistent with improvements in processing-speed related performance and dual-task gait speed, but they do not demonstrate improved inhibitory function or a specific physiological mechanism. However, for single-task gait, although the multivariate analysis revealed a significant group effect, the overall DFA model failed to reach statistical significance, and none of the individual gait parameters differed significantly between groups.

### 4.3 The impact of IMCT on postural control in middle-aged and older adults

This study found no significant changes in any postural-control metrics across the three groups. This negative finding is important given the fall-prevention rationale of the study. Sörlén et al. (2021) likewise reported that a four-week short-term balance-training program failed to reduce COP sway in older adults, supporting our finding of no significant between-group difference. Systematic reviews suggest that balance training must run for at least 11–12 weeks, with ≥3 sessions per week and 36–40 total sessions, to achieve stable reductions in COP sway; the present study involved only a single acute session, which was well below that effective threshold. Additionally, attentional resources captured during a participant’s first exposure to the task may transiently diminish postural stability. Future work should extend the intervention period and include metrics such as dynamic stability boundaries, obstacle negotiation, and reactive step-length adjustments to more sensitively detect fall-related changes. Therefore, the present dual-task gait findings should be interpreted as improvements in cognitive-motor walking performance rather than direct evidence of reduced fall risk.

Although this finding is gait-related rather than a direct postural-control outcome, changes in dual-task double-support time are closely related to walking speed; generally, there is an inverse relationship between walking speed and double-support time—as walking speed increases, double-support time decreases, and conversely, slower walking speeds result in longer double-support time (Kettlety et al., 2023). In this study, participants who engaged in motor-cognitive training with a moderate cognitive load showed increased dual-task walking speed and reduced dual-task double-support time likely indicating that they walked more efficiently and were able to complete pace transitions within shorter durations, thereby achieving a smoother walking process.

The effectiveness of the IMCT approach in reducing the magnitude of DTC may be attributed to its multifaceted demands, which align with findings from previous studies. Dual-task training, as evidenced by Plummer-D’Amato et al. (2012) and Schneider et al. (2021), may encourage adaptation to multitasking demands, thereby supporting EF and attentional resources—key factors for older adults who often face challenges in these areas, leading to increased fall risk and decreased functional independence. By incorporating complex cognitive tasks that require problem-solving or memorization, as suggested by Hunter et al. (2018) and Fallahtafti et al. (2021), the training can yield greater improvements in dual-task performance, as these tasks more effectively challenge cognitive resources. Neuroimaging studies in related contexts further suggest that effective dual-task performance is linked to interactions between the cerebral cortex and cerebellum, which may facilitate the integration of cognitive and motor tasks (Müller et al., 2023; Wu et al., 2013). These neural interactions were not measured in the present study.

It is important to note that during dual-task walking, older adults may prioritize cognitive tasks at the expense of walking stability. This shift in priorities can be influenced by both the nature of the cognitive task and the specific walking conditions. For instance, when older adults are instructed to concentrate on cognitive tasks, they demonstrate greater DTC in terms of gait speed and variability (Ali et al., 2025; Pichierri et al., 2011). In this study, as dual-task walking speed improved most under medium cognitive load, low cognitive load was associated with more favorable changes in calculation attempts and correct responses. Cognitive accuracy itself did not show a significant corrected group effect. This suggests that the enhancements facilitated by IMCT may reflect improved cognitive-motor coordination rather than a simple shift in priorities, although task-prioritization effects cannot be ruled out.

## 5 Limitations

Several limitations should be acknowledged. First, the sample size was relatively small and consisted of middle-aged and older adults recruited from a limited community setting, which may limit generalizability. Second, the motor-cognitive dual-task used in this study primarily focused on working memory, spatial orientation, and logical calculation, while other cognitive domains were not specifically targeted. Third, this was a single-session acute protocol; therefore, the long-term effects of IMCT on EF, dual-task performance, and postural control under varying cognitive loads remain uncertain. Fourth, no non-intervention or active-control group was included, which limits causal interpretation and prevents separation of intervention effects from practice, learning, or repeated-testing effects. Additionally, the assessments were conducted within approximately 1 h after the training session, which further raises concern about potential practice effects. Fifth, although cognitive-load levels were validated with NASA-TLX and task performance before the intervention, NASA-TLX ratings and on-line task accuracy were not re-measured during the 30-min training session itself. Therefore, the assumption that participants experienced sustained low, moderate, and high load during the intervention rests on pre-intervention calibration rather than continuous in-session verification.

Future studies should recruit larger and more diverse samples, include appropriate non-intervention and active-control conditions, and extend the follow-up duration to assess the stability and broader applicability of these findings. Future research should also incorporate a wider range of cognitive tasks and embed brief in-session NASA-TLX, task-accuracy, or psychophysiological probes to verify cognitive load during training. Because the present study combined a single acute intervention with no non-intervention or active-control group, the results are best interpreted as immediate within-session responses rather than as evidence of training-related adaptation.

## 6 Conclusion

In conclusion, this RCT highlights the beneficial effects of appropriate cognitive load on EF and dual-task performance during IMCT. The findings indicate that IMCT, tailored with varying cognitive loads, yields superior outcomes in their respective domains. Specifically, moderate cognitive load was associated with greater improvement in Stroop neutral reaction time, dual-task gait velocity, and DTC magnitude, whereas low cognitive load was associated with greater gains in correct responses during dual-task walking. The DFA results further indicate that Stroop neutral reaction time, 2-back accuracy, gait velocity, and correct responses were relatively sensitive to load differences. These findings may assist practitioners in selecting suitable cognitive-load levels for IMCT, while emphasizing that the present single-session protocol did not produce improvements in static postural control. Therefore, the findings do not directly demonstrate fall-risk reduction, and stronger inferences regarding fall prevention will require longer multi-session interventions that incorporate dynamic balance outcomes and appropriate control conditions.

## Data Availability

The original contributions presented in the study are included in the article/Supplementary material, further inquiries can be directed to the corresponding authors.

## Footnotes

1 “Speed Memory” refers to exercises in which participants briefly view one or more target fields, remember their locations or sequences, and then run to them after the visual cue disappears.

2 “Speed Brain” refers to a category of cognitively demanding exercises that combine rapid movement with mental tasks such as matching pairs, color-word identification, spelling, math solving, and quiz answering.
