# Supplementary material for: Effects of cognitive workload during motor-cognitive training on executive function, dual-task performance, and posture control: a RCT among middle-aged and older adults
Source: Front Aging Neurosci. 2026 Aug 5;18:1861129. doi: 10.3389/fnagi.2026.1861129 (PMC13486162; doi:10.3389/fnagi.2026.1861129)
Supplement: Supplementary file 1 [file Data_Sheet_1.DOCX]

Supplementary Material

**Supplementary Figure 1.** Canonical discriminant function group centroid plot of executive functions.

**Supplementary Table S1.** Pre-to-post change scores (Δ), 95% confidence intervals, and percentage changes for executive function outcomes by cognitive load group (N = 42).

| **Variable** | **LCG (n=14)** | | | **MCG (n=14)** | | | **HCG (n=14)** | | |
| --- | --- | --- | --- | --- | --- | --- | --- | --- | --- |
|  | **Δ ± SD** | **95% CI** | **% change** | **Δ ± SD** | **95% CI** | **% change** | **Δ ± SD** | **95% CI** | **% change** |
| Stroop incongruent accuracy (%) | 0.08 ± 0.13 | [0.01, 0.16] | +9.5% | 0.11 ± 0.09 | [0.06, 0.16] | +12.6% | 0.07 ± 0.12 | [-0.00, 0.14] | +8.0% |
| Stroop neutral accuracy (%) | 0.03 ± 0.02 | [0.02, 0.04] | +3.1% | 0.03 ± 0.03 | [0.01, 0.05] | +2.8% | 0.04 ± 0.06 | [0.00, 0.07] | +4.0% |
| Stroop incongruent RT (ms) | -175.31 ± 203.11 | [-292.58, -58.04] | -13.2% | -248.91 ± 115.84 | [-315.80, -182.03] | -18.4% | -149.64 ± 143.30 | [-232.37, -66.90] | -11.8% |
| **Stroop neutral RT** (ms) | -209.33 ± 160.74 | [-302.14, -116.52] | **-18.0%** | -413.55 ± 162.08 | [-507.13, -319.97] | **-34.8%** | -86.94 ± 78.73 | [-132.40, -41.48] | **-7.0%** |
| MoS non-switching accuracy (%) | 0.03 ± 0.07 | [-0.01, 0.07] | +2.9% | 0.03 ± 0.08 | [-0.02, 0.07] | +2.9% | 0.03 ± 0.07 | [-0.01, 0.07] | +3.5% |
| MoS switching accuracy (%) | 0.06 ± 0.09 | [0.01, 0.11] | +7.0% | 0.06 ± 0.09 | [0.01, 0.11] | +6.7% | 0.04 ± 0.08 | [-0.01, 0.08] | +4.2% |
| MoS non-switching RT (ms) | -105.46 ± 110.87 | [-169.48, -41.44] | -12.0% | -92.52 ± 71.81 | [-133.98, -51.06] | -10.5% | -45.99 ± 52.31 | [-76.19, -15.78] | -5.4% |
| MoS switching RT (ms) | -82.70 ± 119.51 | [-151.71, -13.70] | -7.8% | -126.85 ± 131.78 | [-202.94, -50.76] | -11.6% | -87.99 ± 128.36 | [-162.10, -13.88] | -8.0% |
| 2-back RT (ms) | 18.60 ± 248.93 | [-125.13, 162.33] | +1.8% | -75.50 ± 253.29 | [-221.74, 70.75] | -6.6% | -64.88 ± 226.38 | [-195.59, 65.82] | -5.8% |
| 2-back accuracy (%) | 0.15 ± 0.15 | [0.06, 0.24] | +26.2% | -0.00 ± 0.25 | [-0.15, 0.14] | -0.5% | -0.03 ± 0.17 | [-0.12, 0.07] | -3.9% |

Note. Δ = post-minus-pre change score; values are mean ± SD. % change = mean Δ relative to baseline mean. LCG = low cognitive load group; MCG = medium; HCG = high.

**Supplementary Figure 2**. Canonical discriminant function group centroid plot of dual-task gait performance.

**Supplementary Table S2.** Pre-to-post change scores (Δ), 95% confidence intervals, and percentage changes for dual-task gait and cognitive-performance outcomes by cognitive load group (N = 42).

| **Variable** | **LCG (n=14)** | | | **MCG (n=14)** | | | **HCG (n=14)** | | |
| --- | --- | --- | --- | --- | --- | --- | --- | --- | --- |
|  | **Δ ± SD** | **95% CI** | **% change** | **Δ ± SD** | **95% CI** | **% change** | **Δ ± SD** | **95% CI** | **% change** |
| **DT gait velocity (**m/s**)** | 0.09 ± 0.06 | [0.05, 0.12] | **+12.0%** | 0.26 ± 0.16 | [0.17, 0.35] | **+36.3%** | 0.13 ± 0.11 | [0.07, 0.20] | **+19.6%** |
| DT step length **(**cm**)** | 0.17 ± 2.56 | [-1.31, 1.65] | +0.3% | 1.38 ± 5.06 | [-1.55, 4.30] | +2.7% | -0.46 ± 3.33 | [-2.38, 1.46] | -0.9% |
| DT stride length **(**cm**)** | 1.15 ± 7.09 | [-2.95, 5.25] | +1.0% | 2.11 ± 11.49 | [-4.53, 8.74] | +2.0% | 0.16 ± 6.64 | [-3.67, 3.99] | +0.1% |
| **DT double-support time (**s**)** | 0.03 ± 0.12 | [-0.04, 0.10] | **+6.5%** | -0.14 ± 0.13 | [-0.21, -0.06] | **-24.4%** | -0.04 ± 0.13 | [-0.12, 0.03] | **-7.3%** |
| DT propulsion phase **(**%**)** | 0.15 ± 2.97 | [-1.57, 1.87] | +0.2% | 1.08 ± 6.33 | [-2.58, 4.73] | +1.8% | 0.14 ± 4.73 | [-2.59, 2.87] | +0.2% |
| DT double-support phase **(**%**)** | -0.11 ± 2.92 | [-1.79, 1.58] | -0.4% | -1.45 ± 6.88 | [-5.42, 2.52] | -4.4% | -0.64 ± 5.34 | [-3.72, 2.45] | -2.0% |
| DT step length CV **(**%**)** | -0.95 ± 6.10 | [-4.47, 2.57] | -4.3% | -0.25 ± 9.02 | [-5.46, 4.96] | -1.2% | 0.29 ± 6.10 | [-3.23, 3.81] | +1.5% |
| DT double-support CV **(**%**)** | -3.78 ± 15.83 | [-12.92, 5.36] | -9.8% | -8.03 ± 26.99 | [-23.61, 7.56] | -23.7% | -10.26 ± 23.95 | [-24.09, 3.57] | -24.8% |
| DT propulsion phase CV **(**%**)** | -7.60 ± 55.22 | [-39.48, 24.28] | -7.0% | -13.41 ± 44.29 | [-38.99, 12.16] | -15.4% | -1.46 ± 43.39 | [-26.52, 23.59] | -1.6% |
| **Calculation attempts (**n**)** | 3.64 ± 5.84 | [0.27, 7.01] | **+17.0%** | 1.36 ± 2.56 | [-0.12, 2.84] | **+5.5%** | -2.07 ± 3.93 | [-4.34, 0.20] | **-7.4%** |
| **Correct responses (**n**)** | 4.14 ± 5.67 | [0.87, 7.42] | **+20.8%** | 0.50 ± 2.85 | [-1.14, 2.14] | **+2.1%** | -2.14 ± 3.13 | [-3.95, -0.33] | **-8.2%** |
| **Dual-task cost (**%**)** | 0.06 ± 0.06 | [0.02, 0.09] | **+13.8%** | 0.20 ± 0.15 | [0.11, 0.29] | **+48.6%** | 0.09 ± 0.11 | [0.02, 0.15] | **+19.9%** |
| Cognitive accuracy **(**%**)** | 0.04 ± 0.11 | [-0.02, 0.10] | — | -0.04 ± 0.09 | [-0.10, 0.01] | — | -0.03 ± 0.14 | [-0.11, 0.06] | — |

Note. DT = dual-task.

**Supplementary Table S3.** Pre-to-post change scores (Δ), 95% confidence intervals, and percentage changes for single-task gait outcomes by cognitive load group (N = 42).

| **Variable** | **LCG (n=14)** | | | **MCG (n=14)** | | | **HCG (n=14)** | | |
| --- | --- | --- | --- | --- | --- | --- | --- | --- | --- |
|  | **Δ ± SD** | **95% CI** | **% change** | **Δ ± SD** | **95% CI** | **% change** | **Δ ± SD** | **95% CI** | **% change** |
| ST step length (cm) | -0.57 ± 3.05 | [-2.33, 1.19] | -0.8% | -2.16 ± 2.80 | [-3.78, -0.55] | -3.3% | 3.61 ± 17.44 | [-6.45, 13.68] | +5.6% |
| ST stride length (cm) | 3.62 ± 6.11 | [0.10, 7.15] | +2.8% | 1.39 ± 5.01 | [-1.51, 4.28] | +1.1% | 3.95 ± 20.96 | [-8.15, 16.05] | +3.1% |
| ST double-support time (s) | 0.01 ± 0.03 | [-0.01, 0.03] | +3.9% | -0.00 ± 0.03 | [-0.02, 0.01] | -1.5% | 0.00 ± 0.03 | [-0.02, 0.02] | +0.6% |
| ST propulsion phase (%) | 2.89 ± 6.18 | [-0.68, 6.46] | +5.2% | 0.97 ± 4.52 | [-1.64, 3.58] | +1.7% | -1.01 ± 5.73 | [-4.32, 2.29] | -1.8% |
| ST double-support phase (%) | -2.56 ± 6.28 | [-6.18, 1.07] | -8.4% | -0.47 ± 4.30 | [-2.95, 2.01] | -1.6% | 0.94 ± 5.11 | [-2.01, 3.89] | +3.2% |
| ST step length CV (%) | 0.06 ± 4.99 | [-2.83, 2.94] | +0.8% | -1.53 ± 7.93 | [-6.11, 3.05] | -15.3% | 1.30 ± 4.86 | [-1.51, 4.11] | +19.8% |
| ST double-support CV (%) | 1.52 ± 4.24 | [-0.93, 3.97] | +26.2% | -0.58 ± 2.97 | [-2.30, 1.14] | -8.4% | 1.87 ± 3.77 | [-0.31, 4.05] | +36.5% |
| ST propulsion phase CV (%) | 1.36 ± 8.68 | [-3.64, 6.37] | +9.9% | 7.42 ± 30.04 | [-9.92, 24.76] | +37.5% | -3.69 ± 10.23 | [-9.59, 2.22] | -21.3% |

Note. No single-task gait outcome reached significance after Holm correction. ST = single-task.

**Supplementary Table S4.** Pre-to-post change scores (Δ), 95% confidence intervals, and percentage changes for postural control outcomes by cognitive load group (N = 42).

| **Variable** | **LCG (n=14)** | | | **MCG (n=14)** | | | **HCG (n=14)** | | |
| --- | --- | --- | --- | --- | --- | --- | --- | --- | --- |
|  | **Δ ± SD** | **95% CI** | **% change** | **Δ ± SD** | **95% CI** | **% change** | **Δ ± SD** | **95% CI** | **% change** |
| Eyes-open sway area | 19.41 ± 26.98 | [3.83, 34.99] | +34.3% | 16.28 ± 47.22 | [-10.98, 43.54] | +23.8% | -23.00 ± 75.01 | [-66.31, 20.31] | -24.5% |
| Eyes-open COP path length | 44.09 ± 98.21 | [-12.61, 100.80] | +17.6% | 70.06 ± 159.98 | [-22.31, 162.43] | +25.6% | -35.52 ± 160.48 | [-128.18, 57.14] | -11.9% |
| Eyes-open mean velocity | 0.76 ± 2.70 | [-0.80, 2.32] | +8.4% | 2.36 ± 5.36 | [-0.73, 5.46] | +25.9% | -0.96 ± 4.82 | [-3.74, 1.82] | -9.7% |
| Eyes-open anteroposterior amplitude | 0.56 ± 3.59 | [-1.51, 2.64] | +5.9% | -1.18 ± 4.85 | [-3.98, 1.62] | -9.8% | -1.73 ± 5.72 | [-5.03, 1.58] | -13.5% |
| Eyes-open mediolateral amplitude | 0.92 ± 2.38 | [-0.45, 2.29] | +15.6% | -0.21 ± 2.39 | [-1.60, 1.17] | -3.2% | -0.20 ± 2.50 | [-1.65, 1.25] | -3.1% |
| Eyes-closed sway area | -9.24 ± 87.30 | [-59.64, 41.17] | -6.7% | -15.91 ± 119.54 | [-84.94, 53.11] | -9.9% | -21.54 ± 78.63 | [-66.94, 23.86] | -15.6% |
| Eyes-closed COP path length | -28.49 ± 94.59 | [-83.10, 26.13] | -6.0% | 16.19 ± 204.25 | [-101.75, 134.12] | +2.9% | -80.99 ± 192.13 | [-191.92, 29.94] | -18.2% |
| Eyes-closed mean velocity | -0.96 ± 3.16 | [-2.78, 0.87] | -6.1% | -0.22 ± 5.63 | [-3.47, 3.03] | -1.1% | -2.77 ± 6.39 | [-6.46, 0.92] | -18.7% |
| Eyes-closed anteroposterior amplitude | -0.46 ± 5.66 | [-3.73, 2.81] | -3.0% | -1.29 ± 7.58 | [-5.67, 3.09] | -7.5% | -2.44 ± 6.03 | [-5.92, 1.04] | -14.5% |
| Eyes-closed mediolateral amplitude | -0.32 ± 4.41 | [-2.87, 2.22] | -3.0% | 0.59 ± 3.05 | [-1.17, 2.35] | +5.9% | -0.28 ± 3.13 | [-2.09, 1.53] | -2.9% |

Note. No postural control outcome reached significance after Holm correction

**Supplementary Table S5.** Sensitivity analyses (one-way ANCOVA) examining the influence of educational attainment on outcomes that were significant in the primary analyses (N = 42).

| **Domain** | **Outcome** | **Unadj. F(2,39)** | **Unadj. p** | **Adj. F(2,38)** | **Adj. p** | **ηp²** | **Cov. F(1,38)** | **Cov. p** |
| --- | --- | --- | --- | --- | --- | --- | --- | --- |
| Executive function | **Stroop neutral reaction time** | 19.612 | 0.000 | 19.857 | **<0.001** | 0.511 | 1.842 | 0.183 |
| Dual-task gait | **Dual-task gait velocity** | 8.444 | 0.001 | 8.321 | **0.001** | 0.305 | 0.358 | 0.553 |
| Dual-task gait | **Dual-task double-support time** | 6.372 | 0.004 | 6.376 | **0.004** | 0.251 | 0.755 | 0.390 |
| Dual-task gait | **Calculation attempts** | 6.193 | 0.005 | 6.521 | **0.004** | 0.256 | 1.847 | 0.182 |
| Dual-task gait | **Correct responses** | 8.355 | 0.001 | 8.705 | **<0.001** | 0.314 | 1.669 | 0.204 |
| Dual-task gait | **Dual-task cost** | 6.524 | 0.004 | 6.524 | **0.004** | 0.256 | 0.935 | 0.340 |

Note. Unadj. = unadjusted one-way ANOVA on change scores (primary analysis); Adj. = group effect from ANCOVA after adjusting for educational attainment (years of education); Cov. = effect of the covariate (educational attainment); ηp² = partial eta squared for the adjusted group effect. Single-task gait and postural control domains are not shown because no univariate group effect was significant in the primary analyses; in those domains, educational attainment did not significantly influence any change score (all p > 0.05).
